# Myoelectric Gesture Recognition Based on Multiple Mapping and Deep Neural Network

**DOI:** 10.3390/biomimetics11050344

**Published:** 2026-05-14

**Authors:** Shuolei Yin, Wenjing Huang, Huicao Xie, Yihua Li

**Affiliations:** 1 College of Mechanical and Intelligent Manufacturing, Central South University of Forestry and Technology, Changsha 410004, China; 20231200266@csuft.edu.cn (S.Y.); t20152249@csuft.edu.cn (H.X.); 2 College of Management, Central South University of Forestry and Technology, Changsha 410004, China; yhli@csuft.edu.cn

**Keywords:** myoelectric gesture recognition, feature extraction, multiple mapping, deep neural network

## Abstract

Gesture recognition based on surface electromyography (sEMG) signals typically involves extracting features from the signals and then incorporating recognition models to increase the accuracy of classification. In this paper, drawing on the properties of sEMG signals and statistical principles, we propose a novel feature extraction method called “multiple mapping”, designed to construct a high-performance representation of sEMG signals. The multiple mapping approach incorporates sequential mappings, including the sliding average power, lg (base-10 logarithm) mapping, a linear compression principle, and a sigmoid normalization function. The sliding average power captures the intensity variations in sEMG signals across various channels, allowing differentiation between the activity levels of distinct muscle groups. lg mapping adjusts the distribution of the sEMG signals to improve their uniformity, enhancing feature stability and facilitating comparisons. The linear compression and sigmoid normalization emphasize the signals’ central characteristics while compressing the extremes at both ends. Then, the sEMG signals obtained from multiple mapping are transformed into Sem grayscale maps, which are subsequently processed using the deep neural network ResNet50 for gesture recognition. Extensive experiments on three public datasets were conducted, and the average recognition accuracy was 95.26%, 90.81%, and 96.72%, respectively, while it was 96.8% in self-collected recognition tasks. The results demonstrate that the multiple mapping method significantly improves the feature extraction performance for sEMG-based gesture recognition, offering a promising direction for applications in prosthetic gesture control and muscle–computer interaction.

## 1. Introduction

sEMG signals carry abundant information about muscle activity and movement intention [1], and they have been widely adopted in non-invasive human–computer interaction systems, including robotic hand control [2], wheelchair operation, virtual interaction, and assistive rehabilitation robotics [3,4]. In recent years, sEMG-based myoelectric gesture recognition has often applied deep neural networks to interpret sEMG patterns generated by gesture movements, such as convolutional neural networks (CNNs) [5], the LeNet model [6], long short-term memory (LSTM) [7], and the Resnet50 model [8], which have achieved good performance in electromyography in gesture recognition.

The task of gesture recognition based on sEMG signals falls under the category of multi-class classification problems. Within the overall recognition process, the feature extraction of sEMG signals is a necessary and important step. The feature extraction methods used for sEMG signals typically include time-domain features [9], frequency-domain features [10], and nonlinear features [11]. For instance, MAV features are derived based on signal amplitudes [12], whereas RMS features are calculated in relation to the signal energy [13]. These approaches have improved gesture recognition performance by employing a single feature, but combining features and using feature fusion can better address the issue of insufficient expressiveness in individual features. For example, in a human motion prediction study, Zhou [14] integrated six time-domain and four frequency-domain features across three classifiers, achieving recognition accuracy of 89.5% for 52 different movements. Robinson [15] combined five time-domain features and obtained the highest recognition accuracy of 90.57% in an sEMG-based gesture recognition task. Saeed [16] extracted four time-domain features from six sEMG channels and then applied principal component analysis to reduce the dimensionality. By employing an artificial neural network (ANN), average recognition accuracy of 85.41% was achieved on the NinaPro DB1 dataset. Chen [17] extracted multiple characteristics across both the time and frequency domains using correlations of features, which improved the final recognition performance by 3%. Phinyomark [18] selected seven time-domain and time-scaled features, reorganizing the feature sets using several LDA extension algorithms, and achieved average classification accuracy of 94.2% using a linear discriminant classifier.

The power of sEMG signals can reflect the contraction intensity of the corresponding muscles. In the sEMG signal processing field, reference [19] proposed an sEMG feature extraction method based on the autoregressive power spectrum (ARPS), and it achieved good classification performance in sEMG gesture classification. Reference [20] proposed a dynamic energy model from the perspective of power, which can decode continuous hand movements using a small amount of sEMG data by enabling the quantitative comparison of neuromuscular activation characteristics across different muscles. Moreover, logarithmic transformation has been widely used in the field of biomedical data statistics to improve skewed data distributions. It can reduce the influence of extreme values and more robustly reflect the true characteristics of data through the geometric mean [21]. In sEMG signal research, Gagnat [22] analyzed the relationship between the root mean square normalization of sEMG signals and the muscle activation index in a walking electromyography study of children with cerebral palsy; logarithmic transformation was used to correct the distribution bias of the sEMG data, thereby enhancing the indicators reflecting muscle activation. Khushaba [23] proposed a method for extracting sEMG features based on time-domain spectral moments; it applied logarithmic transformation to enhance feature stability, and the reliability of cross-posture gesture recognition was effectively improved. In addition, the sigmoid function has been widely used in the fields of bioelectrical signal processing and deep learning. In research on sEMG signals, the sigmoid function is often used as an activation function in neural networks. In reference [24], sigmoid was used in LSTM gated units, dynamic convolution, and attention mechanisms to complete temporal feature weighting and information filtering in basketball action recognition; in reference [25], the hyperbolic tangent sigmoid was used as an activation function in artificial neural networks to enhance the feature separability and classification accuracy in single-channel sEMG gesture recognition. In the field of image enhancement, Hassan [26] proposed an image contrast enhancement method based on the sigmoid function, which adaptively adjusted the image brightness and darkness through nonlinear mapping, significantly improving the detail visibility and boundary clarity of low-contrast images. This method effectively stretched the dynamic range and improved the overall visual quality of images by performing sigmoid nonlinear normalization on grayscale values.

In this study, the average power, lg mapping, and sigmoid normalization are introduced into the processing of sEMG signals, and we propose a new feature extraction method called multiple mapping. This method constructs features based on the characteristics of sEMG signals and statistical principles. Firstly, the sliding average power is applied to capture the muscle activation intensity differences across various sEMG signal channels. Next, the lg function is mapped to the average power, making the distribution of average power more uniform and balanced, so singular values can be eliminated to render the activation levels of different muscles more distinguishable. Finally, the principle of linear compression and the sigmoid normalization function are employed; the main characteristics in the central sections of the sEMG signals are emphasized, while values at both extremes are compressed. Then, grayscale maps are generated from the sEMG signals obtained through multiple mapping, which are then combined with the deep neural network ResNet50 to perform gesture recognition. In gesture recognition experiments, the method is evaluated using public datasets and acquisition data. The effectiveness of multiple mapping for feature extraction in gesture recognition is validated and compared with the results reported by other researchers.

The rest of the paper is structured as follows: Section 2 describes the feature construction process via multiple mapping and the entire recognition process; Section 3 analyzes the contributions of each step of the multiple mapping method and presents gesture recognition experiments on datasets to assess the model’s performance; Section 4 discusses the results; and Section 5 concludes the paper.

## 2. Materials and Methods

### 2.1. sEMG Signal Acquisition and Denoising

#### 2.1.1. Public Datasets

The public datasets NinaPro DB2, NinaPro DB3, and NinaPro DB6 are used in this paper.

The DB2 dataset was acquired using Trigno Wireless active dual-differential wireless electrodes, and a total of 40 intact subjects (29 males and 11 females) were recruited, with the considered subject characteristics including age, height, weight, and handedness. Their age range was 21–67 years. The data consisted of multimodal data including sEMG, inertial, kinematic, and force signals, covering 49 hand movements plus a rest position. They were divided into three main categories: the first category (E1) included eight uniform opening and closing gestures with the same duration and nine wrist-based gestures; the second category (E2) included twenty-three grasping and functional gestures; the third category (E3) included nine different finger force modes. sEMG signals were sampled at a frequency of 2000 Hz.

The DB3 dataset was collected using twelve Cometa wireless electrodes, with data coming from 11 patients who underwent radial artery amputation. The dataset comprised 52 gestures, categorized into three major groups: the first group (E1) included 12 basic finger movements; the second group (E2) included 17 isometric and isotonic hand gestures as well as basic wrist movements; and the third group (E3) included 23 grasping and functional gestures. During the experimental collection process, each gesture of the subjects was repeated six times, lasting for 5 s each time, with a 3 s break in between, at a sampling frequency 2000 Hz.

The DB6 dataset, focused on the repeatability of sEMG grasp classification, was collected from 10 intact subjects (7 males and 3 females, including 1 ambidextrous subject) aged 21–36 years. It used 14 Delsys Trigno double-differential sEMG wireless electrodes, which were equally spaced around the forearm (8 at the radio humeral joint height and 6 below) and fixed with adhesive bands and hypoallergenic elastic bands. The dataset included 7 grasp types performed on 14 objects, with each grasp repeated 12 times, twice a day, for 5 days. The sEMG signals were sampled at 2000 Hz.

**Figure 1:**
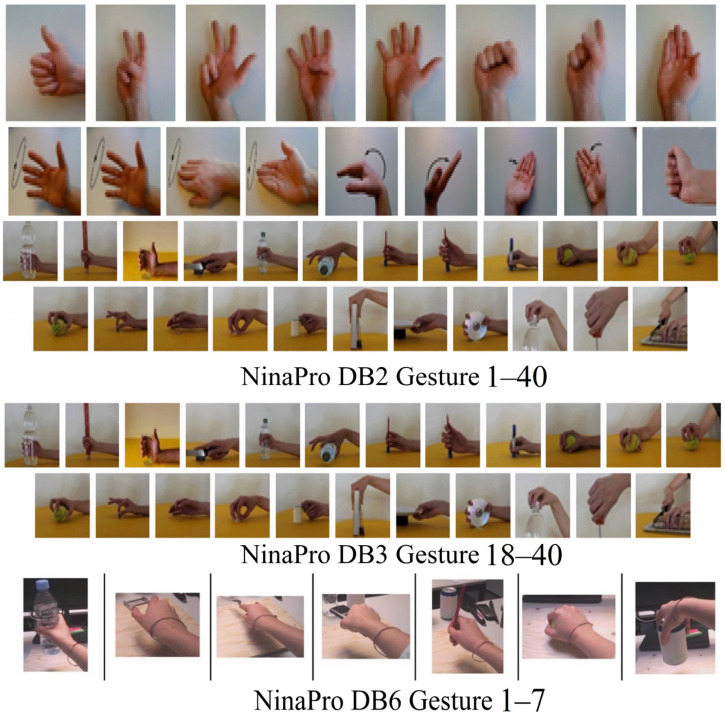
Hand gestures involved in the experiment, derived from public datasets.

The hand gestures selected from the three public datasets are shown in Figure 1.

#### 2.1.2. Dataset Acquisition from Air-Band sEMG Bracelet

**Figure 2:**
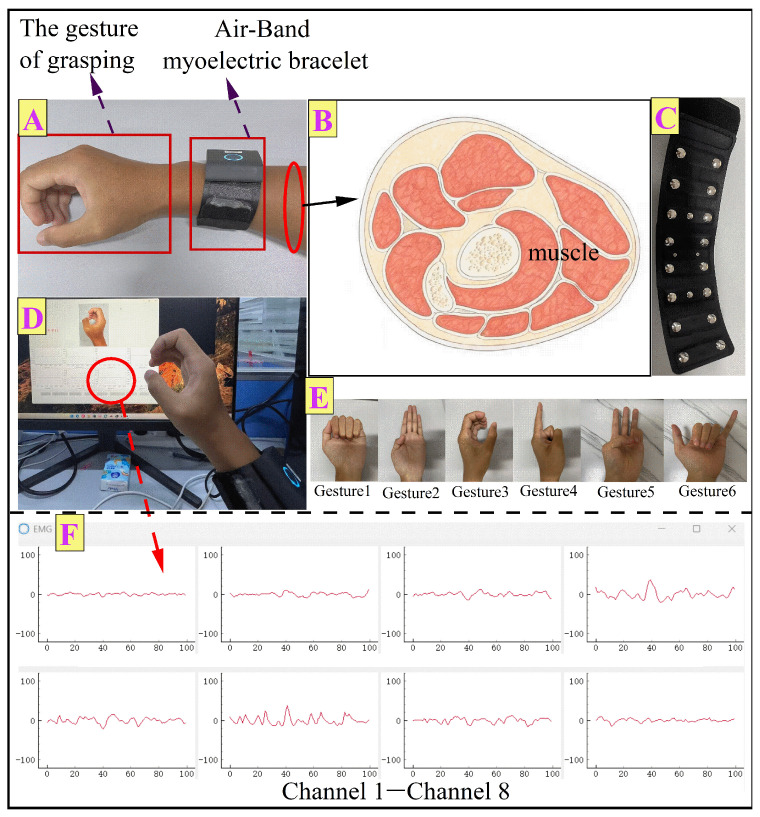
Experiments using Air-Band sEMG bracelet. (**A**) Grasping gesture using Air-Band sEMG bracelet. (**B**) Transverse section of wrist muscles. (**C**) Air-Band sEMG bracelet. (**D**) Experimental scene diagram. (**E**) Hand gestures involved in experiments. (**F**) sEMG signals of 8 channels.

The Air-Band sEMG Wrist Band from Zooeling Technology Co., Ltd. (No. 25, Room 2503, Xiaoshan District, Hangzhou, Zhejiang Province, China) was selected as the sEMG capture device in this study. This sEMG wristband has a strap-type, one-piece modular design and is about 19 cm in width and around 3.5 cm in thickness. Leather fabric is used in the outer part to provide comfort during use, as shown in Figure 2A–C. The wristband uses metallic electrodes to integrate 3 sets of eight electrodes, providing an eighth channel, and has a sampling rate of 1000 Hz. It is water-resistant and integrated with a built-in vibration feedback unit. The wristband has accurate, intrinsically integrated IMU modules to record movement data. It can operate for more than five hours under normal conditions, last seven days without power, and support magnetic induction chargers. The device is equipped with a data visualization interface for acquisition that shows the continuous recording cycle and active-recording gesture details, Ithan instant data output of 8 channels, as shown in Figure 2D,F. Six gestures were captured for each subject, which were as follows: gesture 1, fist-making; gesture 2, four-finger spreading; gesture 3, grasping; gesture 4, index finger spreading; gesture 5, three-finger spreading; gesture 6, shaka. The hand gestures are shown in Figure 2E.

Noise contamination often occurs in sEMG signals, including three types: industrial-frequency interference, Gaussian white noise, and other forms of disturbance and baseline wandering. These factors may reduce the quality of information acquisition to a degree. Improving sEMG analysis requires preprocessing. The amplitude range of the sEMG signals output by electrodes generally falls within 15~100 μV. As the power density was predominantly concentrated in the frequency interval of 10 Hz to 500 Hz, a fourth-order Butterworth low-pass filter with the pass-band edge set at 10 Hz and a cutoff of 500 Hz was selected.

### 2.2. Feature Extraction and sEMG Feature Image Construction

In this paper, we construct high-performance features of sEMG signals using multiple mapping, including the sliding average power, the lg function, linear compression, and sigmoid normalization. These features are then transformed into grayscale maps and fused with the deep neural network ResNet50 to complete the sEMG gesture recognition task.

**Figure 3:**
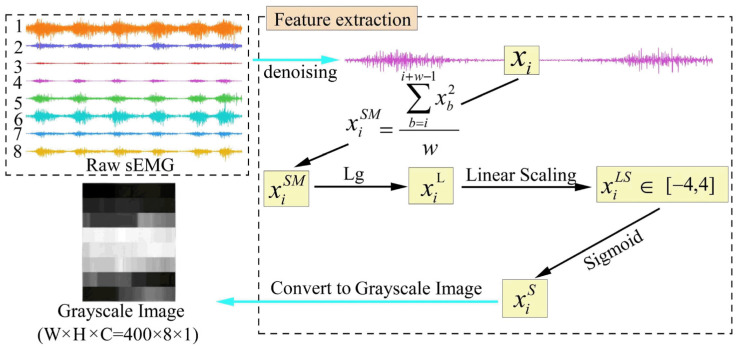
The multiple mapping method.

The original sEMG signals are typically acquired through multiple channels, with each channel reflecting the activity of a specific muscle. Because muscle activation varies in each channel during the execution of a specific gesture, we first apply the sliding average power method to extract inter-channel differences. Next, to amplify the finer details within the signal, the lg function is used to adjust the data distribution, allowing small-scale features to become more distinguishable. Finally, we apply linear scaling followed by sigmoid normalization. This process emphasizes the main characteristics located in the central section of the sEMG signal and compresses values at both extremes. The entire process is illustrated in Figure 3.

#### 2.2.1. Sliding Average Power

When performing a specific gesture, the activity levels of various muscle groups differ, and this variation is reflected in the sEMG signals captured by different channels. To analyze these inter-channel differences in muscle activity, we propose the sliding average power method.

**Figure 4:**
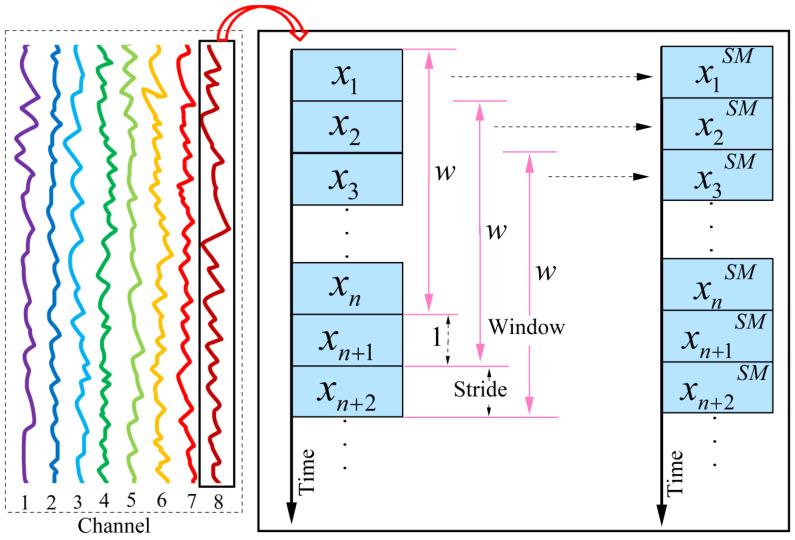
The sliding average power window.

sEMG signals are discrete signals with a finite length, and their average power over a given time period reflects the signal’s strength during that time. To maintain continuity across the signal and avoid data loss after computing the average power, we first slide the sEMG signals using a fixed window length w, with the sliding stride set to 1 data point. For each window, we calculate the average power of the corresponding segment of the sEMG signal. The formula used is presented in Equation (1):

(1) $$x_{i}^{SM}=\frac{\sum_{b=i}^{i+w-1}{x_{b}}^{2}}{w}$$

where $x_{i}$ denotes the ith data of the channel, $x_{i}^{SM}$ denotes the calculated average power value, and i+w−1 means that w data were taken from $x_{i}$, i=1,2,3…,n. The sliding average power calculation process is shown in Figure 4.

#### 2.2.2. lg Mapping

The time-domain amplitude of the raw sEMG signal generally fluctuates around $10^{-5}$, so most of them are located in the range $\left[10^{-6}\right.,\left.10^{-4}\right]$. After applying the sliding average power method in the previous step, the magnitude of the signal shows significant variation. The amplitude distribution within and across channels often becomes approximately skewed. If these skewed data are directly normalized, a large portion may become compressed within a narrow region of the normalization function, leading to inevitable feature loss and a drop in classification performance. Thus, we apply logarithm mapping to the signal data obtained from the previous step. Logarithm mapping can reduce the dynamic range of the sEMG signals and reveal fine details that are easier to compare and analyze.

We assume the sEMG signal to be a random variable X. Assuming that it follows a lognormal distribution, the probability density function of the random variable X is shown as follows (2):

(2) $$f(X)=\frac{1}{\sqrt{2\pi }\sigma X}\cdot e^{-{\left(\ln X-\mu \right)}^{2}/2\sigma^{2}},X>0$$

where μ is the mean of X, μ is the variance of X, and σ is the standard deviation. Taking the logarithm of the base 10 for the random variable X, the probability density function of the random variable $Y=\log_{10}^{X}$ is

(3) $$\begin{array}{l} f(y)={\left(10^{y}\right)}^{\prime }\cdot \frac{1}{\sqrt{2\pi }\cdot \sigma \cdot 10^{y}}\cdot e^{-\frac{{\left[\ln\left(10^{y}\right)-\mu \right]}^{2}}{2\sigma^{2}}}\\ =10^{y}\cdot \ln10\cdot \frac{1}{\sqrt{2\pi }\cdot \sigma \cdot 10^{y}}\cdot e^{-\frac{{\left[\ln\left(10^{y}\right)-\mu \right]}^{2}}{2\sigma^{2}}}\\ =\frac{\ln10}{\sqrt{2\pi }\cdot \sigma }\cdot e^{-\frac{{\left[\ln\left(10^{y}\right)-\mu \right]}^{2}}{2\sigma^{2}}}\\ =\frac{\ln10}{\sqrt{2\pi }\cdot \sigma }\cdot e^{-\frac{{\left(y-u/\ln10\right)}^{2}}{{\left(\sqrt{2}\sigma /\ln10\right)}^{2}}} \end{array}$$

It can be seen that $Y=\log_{10}^{X}$ is an approximately normally distributed random variable if X is a random variable following a skewed distribution. In other words, base-10 logarithmic transformation can adjust the skewed distribution of the sEMG signals toward a more normal distribution, making the overall distribution more even and balanced. It also reduces the impact of extreme values.

For these reasons, we adopt lg (base-10 logarithm) mapping and apply it to the sEMG data obtained through sliding average power processing. Moreover, we take the absolute value of the result to ensure that all values remain positive. The computation is shown in Equation (4):

(4) $$x_{i}^{L}=\left|\mathrm{lg}x_{i}^{SM}\right|$$

where $x_{i}^{SM}$ is the sEMG signal obtained from sliding average power processing, and $x_{i}^{L}$ is the sEMG signal after lg mapping.

#### 2.2.3. Sigmoid Normalization

The sigmoid function is an “S”-shaped curve. Its two ends are relatively flat, allowing it to compress values at both extremes and reduce the influence of outliers in sEMG signals. In contrast, the central region of the sigmoid function changes rapidly, so, when the signal is normalized, its main data points are mapped to this middle section, which helps to emphasize the fine details of the signal. Based on this characteristic, we select the sigmoid function as the normalization method.

The normalization process begins with the linear compression of the sEMG signals obtained from the lg mapping step. This linear scaling step adjusts the data range without changing their distribution. The formula for linear scaling is shown in Equation (5):

(5) $$x_{i}^{LS}=\left[J\left(x_{i}^{L}-x_{i_{\min}}^{L}\right)/\left(x_{i_{\max}}^{L}-x_{i_{\min}}^{L}\right)\right]-J/2$$

where $x_{i}^{L}$ is the sEMG signal obtained from lg mapping processing, and $x_{i}^{LS}$ is the sEMG signal obtained from linear scaling. $x_{i_{\min}}^{L}$ denotes the minimum value in $x_{i}^{L}$, and $x_{i_{\max}}^{L}$ denotes the maximum value in $x_{i}^{L}$. J is calculated as in Appendix A.

Next, the sigmoid function operation is performed on the data $x_{i}^{LS}$ obtained from linear compression. The sigmoid function expression is shown in Equation (6):

(6) $$x_{i}^{S}=\frac{1}{1+e^{-x_{i}^{LS}}}$$

where $x_{i}^{LS}$ is the data obtained from the linear scaling process, and $x_{i}^{S}$ is the data obtained from sigmoid normalization.

#### 2.2.4. Grayscale Image

**Figure 5:**
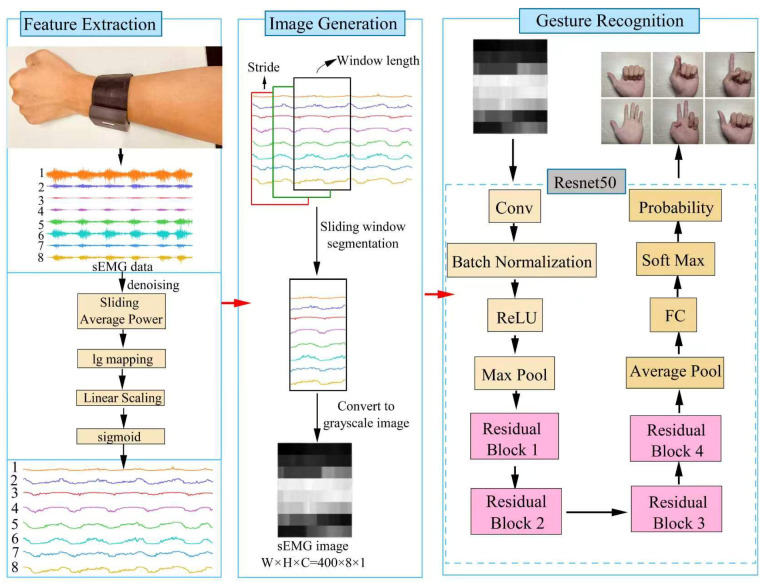
Gesture recognition progress based on multiple mapping and deep neural network.

After the application of multiple mapping as described above, we obtain 8 rows of feature data for the sEMG signals, which correspond to 8 channels in sequence, as shown in Figure 5. The feature data are used to generate grayscale maps. We adopt a sliding window approach to process the data sequentially. The stride length is h. The feature data are segmented into small fragments. For each fragment, L is the data amount from each channel, T is the number of acquisition channels, and L is calculated as shown in Equation (7):

(7) $$L=\frac{k\times f}{1000}$$

where k is the length of the sliding window, and f is the sampling frequency of the sEMG signals. Meanwhile, 1000 is the unit conversion factor, converting seconds to milliseconds (1 s is 1000 ms).

Then, assuming that the value range of $x_{i}^{S}$ obtained from sigmoid normalization is $\left[x_{i\min}^{S},x_{i\max}^{S}\right]$, the calculation expression of the gray value y is as given by Equation (8):

(8) $$y=255\cdot \frac{x_{i}^{S}-x_{i_{\min}}^{S}}{x_{i_{\max}}^{S}-x_{i_{\min}}^{S}}$$

where $x_{i_{\min}}^{S}$ denotes the minimum value in $x_{i}^{S}$, and $x_{i_{\max}}^{S}$ denotes the maximum value in $x_{i}^{S}$. In this way, the grayscale image is formed, as shown in Figure 3.

### 2.3. Gesture Recognition Based on Multiple Mapping and Deep Neural Network

Grayscale maps are generated from the sEMG signals obtained through multiple mapping; then, these grayscale maps are combined with the deep neural network ResNet50 to perform gesture recognition. ResNet50 is a 50-layer deep residual network composed of convolutional layers, pooling layers, residual blocks, and fully connected layers. The architecture of ResNet50 and the process of gesture recognition based on multiple mapping and deep neural networks are illustrated in Figure 5.

As shown in Figure 5, the gesture recognition process is divided into three main stages. In the first stage, feature extraction is carried out by performing multiple mapping on the raw sEMG signals. In the second stage, grayscale maps are generated by applying the sliding window technique to the processed signals. In the third stage, the generated grayscale images are input into ResNet50 to perform gesture classification.

## 3. Results

In this study, we use the gesture recognition accuracy and average recognition accuracy as evaluation metrics. The gesture recognition accuracy (accuracy) for each subject is defined as the ratio between the number of correctly classified gestures and the total number of predicted gestures. The average recognition accuracy (average accuracy) is defined as the average value of the recognition accuracy.

The training device is equipped with an I5-12600KF CPU and an RTX4060 GPU. All experiments use a sliding window of 200 ms with a step size of 50 ms to transform the processed data into grayscale maps. The initial learning rate of ResNet50 is set to $10^{-5}$.

### 3.1. Validity Analysis

The validity analysis is conducted using the publicly available NinaPro DB2 dataset.

#### 3.1.1. Sliding Average Power

**Figure 6:**
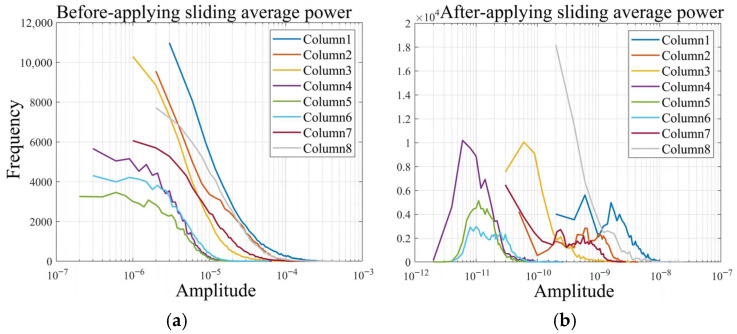
The amplitude variations of the sEMG signals before and after being processed with the sliding average power. (**a**) The amplitude variations of the sEMG signals before sliding average power processing; (**b**) the amplitude variations of the sEMG signals after sliding average power processing.

The purpose of the sliding average power is to represent the activity level of each sEMG signal channel using the average power, allowing for clearer distinction between channels. Since sEMG signals typically appear 50–100 ms before actual movement begins, we set the sliding window length *w* to 200, corresponding to a 100 ms duration, to obtain a more accurate representation of the signal activity. We analyze gesture 1 for five subjects (S1–S5) in the DB2 dataset. Figure 6 shows the results of the comparison of the sEMG signal distribution of one subject before and after applying the sliding average power; it presents the first 70% of the signal data from channels 1 to 8.

As seen in Figure 6a, the data distribution of the original sEMG signal of each channel has a high degree of overlap. The signal amplitude is mainly concentrated around $10^{-5}$, most of them are located in the range $\left[10^{-6}\right.,\left.10^{-4}\right]$, and the difference in activity between different channels is not significant, despite the fact that there is a frequency difference in each channel at different amplitudes. After the sliding average power processing, as shown in Figure 6b, the amplitude of the sEMG signal of each channel aggregates toward different orders of magnitude, and the amplitude distributions of some channels are completely free of overlapping regions, so that the activity differences between different channels are obvious and there is a large degree of differentiation.

As seen in Figure 6b, after processing the sEMG signals with the average power, most of them are located in the range $\left[10^{-12}\right.,\left.10^{-8}\right]$, and channels with more intense muscle activity have greater amplitudes corresponding to their signals. The amplitudes of the channel 1 and 8 data for subject S1 were concentrated near $10^{-9}$ when subject S1 completed gesture 1, indicating that the corresponding muscle group was characterized by vigorous activity. The amplitudes of the data for channels 2, 3, and 7 are concentrated around $10^{-10}$, corresponding to muscle groups with slightly weaker activity. In contrast, the amplitudes of the data for channels 4, 5, and 6 are concentrated around $10^{-11}$, corresponding to the muscle groups with the weakest activity at the completion of the gesture.

These results confirm that processing sEMG signals with the sliding average power can effectively separate channel-specific activity. This transformation highlights muscle activity differences at the data level and provides meaningful support for gesture classification.

#### 3.1.2. lg Mapping

lg mapping helps to render the signal distribution more uniform and balanced. This prevents excessive data stacking in narrow ranges and allows subtle features of the sEMG signals in each channel to be better represented. To verify this effect, we selected the first 70% of the data from gestures 1–5 of subjects S1–S5 in the DB2, DB3, and DB6 datasets, respectively. The Jarque–Bera test was used to analyze the distribution characteristics of the sEMG data before and after applying lg mapping.

The Jarque–Bera test is commonly used to assess normality in large-sample data. It evaluates the skewness and kurtosis of the sample distribution. The formulas for calculating skewness (SK) and kurtosis (K) are shown in Equation (9):

(9) $$\begin{array}{l} SK=\frac{m}{\left(m-1\right)\left(m-2\right)}\cdot \sum_{d=1}^{m}{\left(\frac{x_{d}-\bar{x}}{s}\right)}^{3}\\ K=\frac{m\left(m+1\right)\cdot {\sum_{d=1}^{m}\left(\frac{x_{d}-\bar{x}}{s}\right)}^{4}}{\left(m-1\right)\left(m-2\right)\left(m-3\right)}-\frac{3{\left(m-1\right)}^{2}}{\left(m-2\right)\left(m-3\right)} \end{array}$$

where m is the number of samples; $x_{d}$ is the dth value; $\bar{x}$ is the mean, $\bar{x}=\left(1/m\right)\cdot \sum_{d=1}^{m}x_{d}$; and s is the sample standard deviation, $s=\sqrt{\sum_{d=1}^{m}{\left(x_{d}-\bar{x}\right)}^{2}/\left(m-1\right)}$. The kurtosis and skewness values were calculated as shown in Table 1.

As shown by the skewness values for DB2 in Table 1, which presents data from one of the subjects, the raw sEMG data for gestures 1–5 displayed an approximately right-skewed distribution (skewness > 0) after sliding average power processing. After applying lg mapping, the skewness values for all gestures dropped significantly and approached zero. This indicates that lg mapping effectively transformed the right-skewed distribution into one that approximated normality, with the signal data becoming more symmetrically distributed around the mean.

The kurtosis values in Table 1 for DB2 further illustrate this effect. After sliding average power processing, the data for gestures 1–5 were heavily concentrated near the mean (kurtosis > 10), and gesture 4 reached an extremely high kurtosis value of 1396.7. This indicates that there was excessive data stacking in a localized area before lg mapping. After lg mapping, the kurtosis values dropped considerably, moving closer to the kurtosis benchmark of 3 for a normal distribution. These results suggest that lg mapping can reduce extreme clustering and spread the signal data more evenly, improving the overall distribution quality. The skewness and kurtosis results shown in Table 1 for DB3 and DB6 present the same characteristics.

The above analysis of skewness and kurtosis indicates that lg mapping can adjust the skewed distribution of sEMG data, bringing it closer to a normal distribution, which can result in a more uniform and balanced signal distribution, allowing the finer features of each channel to become more visible.

#### 3.1.3. Sigmoid Normalization

Before applying sigmoid normalization, linear compression needs to be performed. The time-domain amplitude of the raw sEMG signal generally fluctuates around $10^{-5}$, and most of them are located in the range $\left[10^{-6},10^{-4}\right]$, so the raw sEMG signals typically span a dynamic range of nearly 100-fold. According to Appendix A, the ratio j of the maximum and minimum amplitude values of the raw sEMG signals is $j=z_{\max}/z_{\min}=10^{-4}/10^{-6}=100$.

After the sliding average power process and lg mapping, the j between the maximum and minimum amplitude values is J=2lgj=4 according to Equation (A1). Thus, the sEMG signals are deflated to the symmetric interval $\left[-J/2,J/2\right]$. Considering that there are inevitably values in the sEMG signals that are beyond the range $\left[10^{-6}\right.,\left.10^{-4}\right]$, we expand the interval $\left[-J/2,J/2\right]$ to the interval $\left[-J_{1}/2,J_{1}/2\right]$, where $J_{1}>J$.

By using the sigmoid function for normalization, we seek to ensure that not only is the range of output values within [0,1] but that the left endpoint is as close to 0 as possible and the right endpoint is as close to 1 as possible. Since $1/\left(1+e^{-4}\right)\approx 0.982$, $1/\left(1+e^{4}\right)\approx 0.018$, so we set $J_{1}=4>J(J=2)$; then, the interval $\left[-J_{1}/2,J_{1}/2\right]$ is $\left[-4,4\right]$. As a result, $x_{i}^{LS}$ is finally mapped to the interval $\left[0.018,0.982\right]$ after sigmoid normalization.

### 3.2. Ablation Experiment

To evaluate the effectiveness of the multiple mapping feature extraction method (sliding average power + lg mapping + sigmoid) proposed in this paper for gesture recognition, we conducted an ablation experiment. The experimental strategy was the hold-out method. A sliding window of 200 ms and a stride size of 50 ms were used to generate grayscale maps from the processed sEMG signals. These maps were divided into a training set and a test set at a ratio of 4:1, with no overlapping between the two sets. Finally, the myoelectric gestures were classified by ResNet50.

We selected five subjects from the DB2 dataset for the ablation experiment: S4 (female, 30 years old), S5 (male, 25 years old), S6 (male, 35 years old), S10 (male, 34 years old), and S22 (female, 28 years old). For each subject, 70% of the data from gestures 1–17 were used as the basis for evaluation. The following comparisons were performed in the ablation study: T1—raw sEMG signals processed with Z-score normalization; T2—sliding average power only; T3—sliding average power + lg mapping; T4—sliding average power + lg mapping + sigmoid normalization.

**Figure 7:**
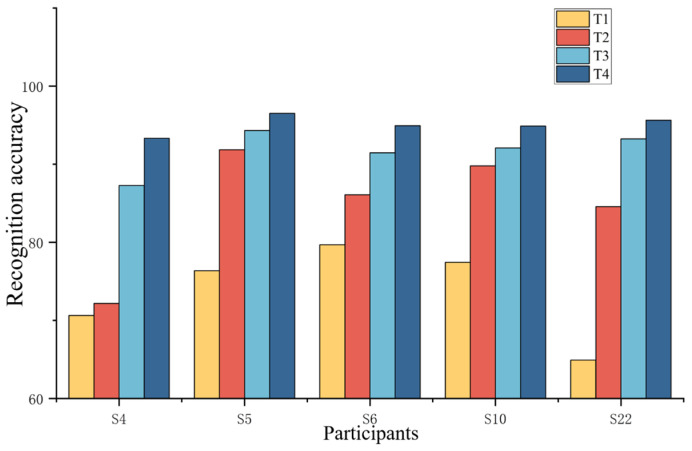
Recognition accuracy for 17 gestures performed by 5 subjects under different experimental conditions. T1 represents experiments under condition T1, T2 represents experiments under condition T2, T3 represents experiments under condition T3, and T4 represents experiments under condition T4.

The results are shown in Figure 7. The experimental results demonstrate a clear improvement in gesture recognition accuracy when introducing the multiple mapping method. The outcomes were as follows. In T1, the raw sEMG signals were Z-score-normalized and converted into grayscale maps before classification with ResNet50. Due to the absence of feature extraction, the 17-gesture recognition across the five subjects achieved average accuracy of 73.81%. In T2, when applying the sliding average power method to extract inter-channel activity differences, a significant improvement was presented, with average accuracy of 84.88%. It was seen that capturing the differences in muscle activity between channels can provide meaningful features for the recognition model and boost its performance. In T3, when applying lg mapping to the sliding average power, the average accuracy reached 91.68%. It was found that adjusting the signal distribution can help to expose finer details in sEMG data, which is beneficial for recognition. In T4, the full multiple mapping method, combining sliding average power, lg mapping, and sigmoid normalization, achieved the highest performance, with average accuracy of 95.05%. This result shows that the multiple mapping method can extract high-quality features by resolving channel-wise differences, improving the data distribution, and compressing extreme values while preserving the signal’s central features.

Overall, the multiple mapping method proposed in this paper can achieve excellent feature extraction from sEMG signals, and its effectiveness was demonstrated in gesture recognition when combined with the deep neural network ResNet 50. It may provide an efficient and accurate solution for gesture recognition based on sEMG signals, with theoretical and practical value.

### 3.3. Gesture Recognition Experiment

#### 3.3.1. Gesture Recognition Based on Public Datasets

In this section, we present gesture recognition on three publicly available datasets: NinaPro DB2, DB3, and DB6. Firstly, the multiple mapping method was used to extract features. After feature construction, the sEMG signals were transformed into grayscale maps, which were then input into the ResNet50 model for gesture recognition. All grayscale maps used in the experiments had a consistent input size. NinaPro DB2 and DB6 were collected from able-bodied subjects, while DB3 was collected from amputees. The dataset selection is summarized in Table 2.

**Figure 8:**
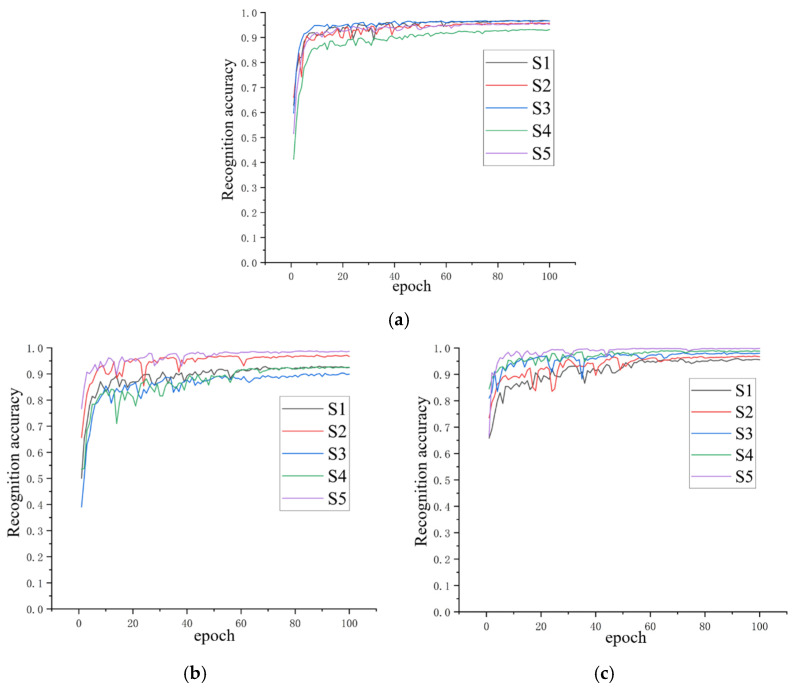
The validation accuracy curves of experiments (hold-out method) on subjects S1–S5 in three datasets (DB2 (**a**), DB3 (**b**), and DB6 (**c**)). The horizontal axis, labeled “epoch”, represents the number of training rounds, and the vertical axis, labeled “validation accuracy”, represents the corresponding accuracy obtained from testing in each training round.

Gesture recognition experiments were evaluated via the hold-out method. The samples were segmented in chronological order. The first 80% of samples served as the training set, while the last 20% served as the validation set. The training set and the test set had no overlapping parts. The training curves of the recognition process are shown in Figure 8.

Figure 8a presents the training curves for the E1 and E2 subsets of the NinaPro DB2 dataset, which together included 40 gestures. It shows the recognition accuracy over 100 validation cycles for five subjects (S1–S5). In the early stages, the recognition accuracy increased rapidly and approached near-peak levels around the 20th cycle, indicating that the model converged quickly during training. After this, the accuracy curves showed small fluctuations and became stabilized. Finally, the recognition accuracy reached approximately 96%. Figure 8b presents the training curves for the E1 subset of NinaPro DB3, which included 17 gestures. It shows the recognition accuracy for subjects S1–S5 across 100 validation cycles. Similarly to the DB2 results, the recognition accuracy increased quickly at the beginning and then remained stable, with minor variation. Figure 8c presents the training curves for the D1-T1 subset of NinaPro DB6, which included seven gestures. It shows the recognition accuracy over 100 validation cycles for subjects S1–S5. The results indicate that the classification performance of all subjects remained stable.

It can be seen that the proposed multiple mapping feature construction method fused with the ResNet50 network maintained reliable and consistent performance in sEMG-based gesture recognition, highlighting its adaptability and stability across different subjects and varying gestures.

The recognition results for NinaPro DB2 (E1, E2), DB3 (E1), and DB6 (D1-T1) are listed in Table 3. The rate distribution in Table 3 reflects the fluctuation range of the recognition accuracy for different subjects. As shown in Table 3, for the 20 subjects in NinaPro DB2, the average recognition accuracy for 40 gestures across the E1 and E2 subsets reached 95.26%, with a variation range of 4.13%. The recognition accuracy remained stable, showing minimal fluctuations. For the 11 subjects in NinaPro DB3, the average recognition accuracy for 17 gestures in the E1 subset was 90.81%, with a larger variation of 22.25%. This wider fluctuation was primarily due to subject S7, who had a complete forearm amputation and no phantom limb sensation (level 0), yielding lower recognition accuracy of 73.21%. For the 10 subjects in NinaPro DB6, the average recognition accuracy for seven gestures in the D1-T1 subset was 96.72%, with a variation range of 2.68%, indicating stable classification results.

**Figure 9:**
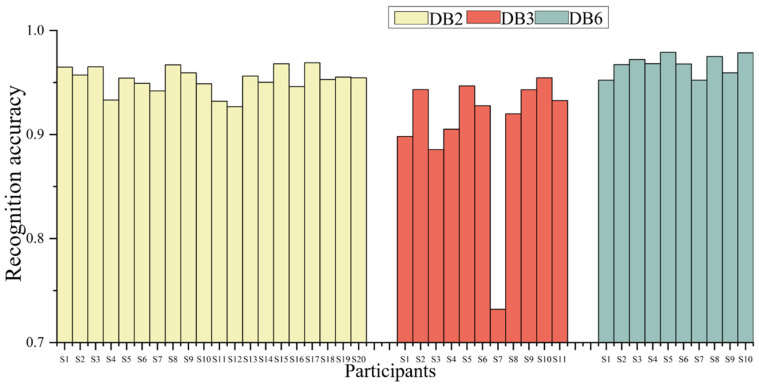
The gesture recognition results based on multiple mapping and a deep neural network.

The results for individual subjects are displayed in Figure 9. As seen in Figure 9, the average recognition accuracy remained consistently high across all three datasets. Within each dataset, the gesture recognition accuracy remained high and stable across different gestures, showing the method’s adaptability. Across the different datasets, the average gesture recognition accuracy also remained stable. For DB3, the average accuracy was 90.81%, with a variation range of 22.25%. DB3 yielded the lowest average accuracy due to its amputee subjects. The accuracy for DB2 was slightly lower than that for DB6, but its recognition fluctuation remained small. These results indicate that the proposed method is capable of adapting to diverse signal characteristics and data conditions.

Then, a comparative analysis was conducted on all three public datasets, NinaPro DB2, NinaPro DB3, and NinaPro DB6. The results were compared with those of mainstream recognition methods reported by other researchers, as shown in Table 4. The results indicated that the proposed method—multiple mapping feature construction fused with the ResNet50 network—outperformed most existing approaches across all three datasets. It showed clear advantages in terms of feature extraction and classification performance.

#### 3.3.2. Gesture Recognition Based on Air-Band sEMG Bracelet

The experimenter wore an Air-Band sEMG bracelet and collected sEMG data on the grasping-mode sEMG signal platform, as shown in Figure 2. It could be connected to a Bluetooth receiver to transmit data in real time to a computer. After receiving the sEMG signals, the receiving platform used a bandpass filter to remove noise from the signals.

We collected six types of gestures on this platform: 1—a fist-making gesture; 2—a four-finger spreading gesture; 3—a grasping gesture; 4—an index finger spreading gesture; 5—a three-finger spreading gesture; 6—shaka. For each gesture, 250 segments of sEMG signals were collected, and they were converted into 250 sEMG grayscale images, resulting in a dataset size of 1500 per experimenter. Five different experimenters were selected, including four male members and one female member. The six gestures were repeated to obtain sEMG signals for each experimenter. The data of each experimenter were used to conduct the experiment. According to the collection time, the data were divided into a training set and a validation set, with a ratio of 80% to 20%, respectively, and there was no overlap between the two sets. The test results are shown in Table 5.

As shown in Table 5, the average recognition accuracy for the five experimenters was 96.8%, indicating that the multiple mapping method combined with the deep neural network ResNet50 can perform well across different individuals. Besides the low gesture recognition accuracy of 92.33% for experimenter S2, the gesture recognition accuracy for all experimenters was relatively high.

**Figure 10:**
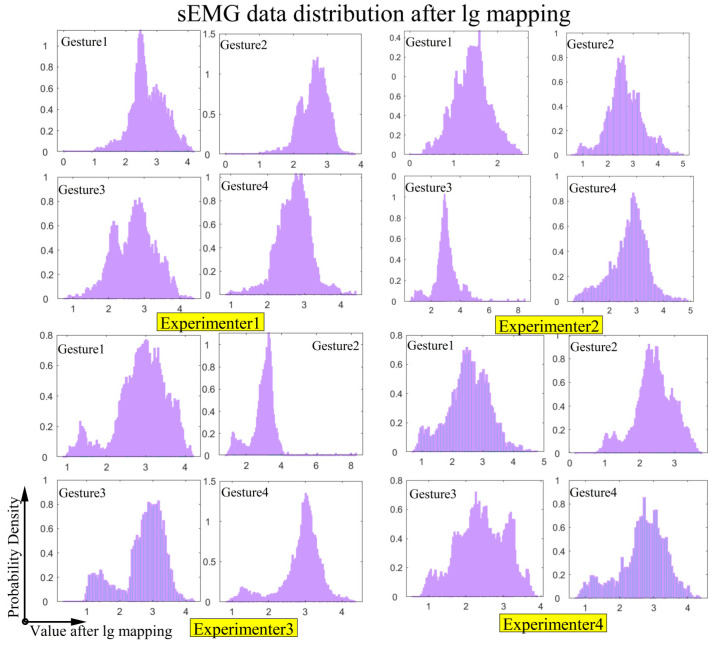
sEMG data distribution for 4 gestures among 4 experimenters after lg mapping.

The distribution of the sEMG signals after lg mapping is shown in Figure 10. It can be seen that the data distribution was approximately to normal and balanced, and the impact of extreme values was reduced by lg mapping, which aided in the learning of the gesture recognition model.

**Figure 11:**
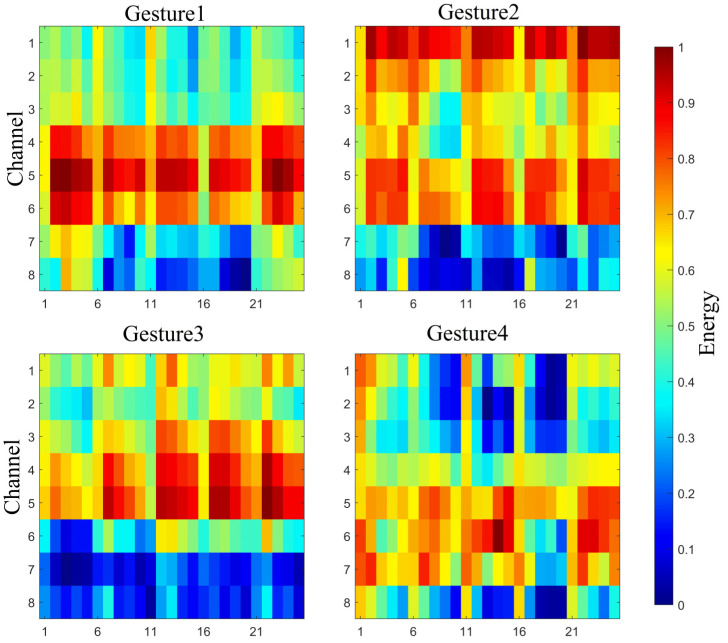
Energy heatmap results for experimenter 1.

The energy heatmaps depicting the results for experimenter 1 are shown in Figure 11. It can be seen that the energy levels of the different channels varied for this experimenter. Since these channels corresponded to different muscles, the muscle activity strength was successfully captured when completing specific gestures. The energy heatmaps of the different gestures presented dissimilar distribution characteristics, corresponding to the distribution of muscle activation levels between channels, so this can assist in gesture classification.

**Figure 12:**
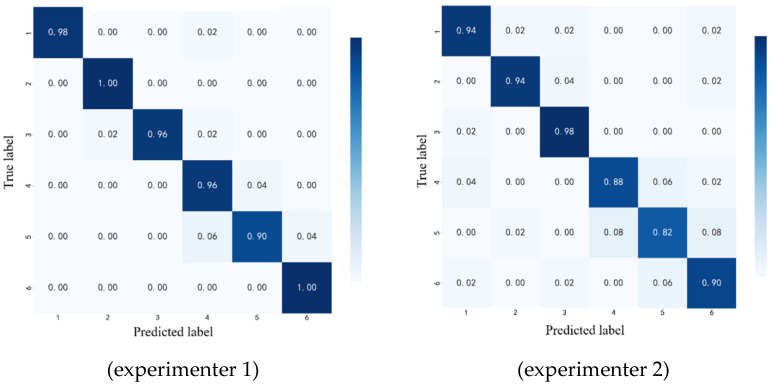

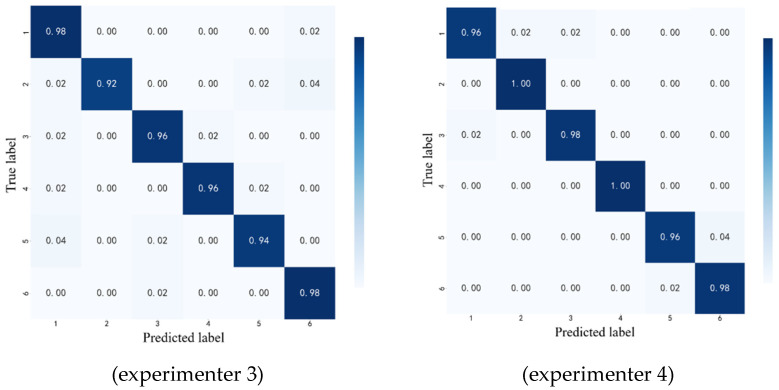
Confusion matrices of gesture recognition results for experimenters 1–4.

The confusion matrices of the gesture recognition results for four of the experimenters are shown in Figure 12. This figure shows that the average recognition accuracy for experimenter 1 was 97.67%, indicating that the gesture recognition results were ideal. For experimenter 2, the average recognition accuracy was 92.33%. It can be seen that the recognition performance for gestures 1, 2, and gesture 3 was ideal, but the recognition performance for gestures 4 and 5 was unsatisfactory, with difficulties in distinguishing between gestures 4, 6, and 5. Figure 12 also shows the confusion matrices for experimenters 3 and 4. For both experimenters, a high level of gesture recognition accuracy was achieved, at 98%, but there were cases of misclassification. For example, for experimenter 3, gesture 2 was easily misidentified as gesture 6, and, for experimenter 4, gesture 5 was easily recognized as gesture 6.

In general, the multiple mapping method has good feature construction performance in sEMG gesture recognition tasks. It has demonstrated good recognition accuracy and stability in human sEMG gesture recognition tasks.

## 4. Discussion

The multiple mapping method proposed in this study constructs features through processing steps including the sliding average power, lg mapping, linear scaling, and sigmoid normalization. These features effectively enhance the accuracy and robustness of sEMG-based gesture recognition. Experimental results from both public datasets and our self-constructed dataset demonstrate the method’s outstanding performance, achieving high gesture recognition accuracy in both able-bodied and amputee subjects. This indicates the method’s suitability for feature extraction from sEMG signals.

The multiple mapping method introduces innovation regarding the characteristics of sEMG signals and statistical data distribution optimization, and it provides a new approach to the processing of sEMG signals. Since the method introduces additional transformations, it increases the preprocessing overhead, so the computational work will be greater. However, according to the experimental results, the average processing times for DB2, DB3, and DB6 were 0.0018 s, 0.0004 s, and 0.0018 s, respectively, per sample data. Thus, this overhead may have little impact on the real-time recognition of sEMG gestures.

## 5. Conclusions

In summary, this work proposes a novel multiple mapping method for constructing sEMG signal features. It includes the sliding average power to resolve inter-channel activity differences, lg mapping to adjust the data distribution, and sigmoid normalization to retain essential features while reducing the influence of outliers. The multiple mapping method combined with the deep neural network ResNet50 was applied to conduct gesture recognition experiments on the NinaPro DB2, DB3, and DB6 datasets. It achieved high recognition accuracy and demonstrated robust performance. Specifically, it reached average accuracy of 96.8% in gesture recognition tasks. In general, the proposed method surpasses the performance of the current mainstream approaches, highlighting its effectiveness in real-world sEMG gesture recognition.

## Figures and Tables

**Table 1:** The skewness and kurtosis when using the sliding average power and lg function.

	Gesture 1		Gesture 2		Gesture 3		Gesture 4		Gesture 5	
	SK	K	SK	K	SK	K	SK	K	SK	K
Skewness and kurtosis when using sliding average power and lg function for DB2										
Sliding Average Power	5.2	48.2	3.3	16.1	2.7	11.6	36.0	1396.7	3.3	16.2
lg	0.2	1.7	0.0	2.0	0.3	2.2	0.0	2.3	0.1	2.3
Skewness and kurtosis when using sliding average power and lg function for DB3										
Sliding Average Power	−0.6	7.2	−1.4	6.7	−1.4	6.1	−1.5	7.2	−1.5	7.2
lg	−0.1	3.6	0.1	3.2	0	3.7	0.3	3.9	0.1	7.4
Skewness and kurtosis when using sliding average power and lg function for DB6										
Sliding Average Power	−1.1	5.8	−1.1	5.5	−1	5.2	−1	5.2	−0.9	5
lg	0.4	3.6	−0.8	4.3	0.6	2.8	−0.8	4.7	0.1	2.3

**Table 2:** Three public datasets.

Dataset			Input Size	Number of Gestures
DB2	S1–S20	E1	400 × 8 × 1	17
		E2		23
DB3	S1–S11	E1		17
DB6	S1–S10	D1-T1		7

**Table 3:** The performance on three different datasets.

Dataset			Input Size	Number of Movements	Average Accuracy (%)	Rate Distribution (%)
DB2	S1–S20	E1	400 × 8 × 1	17	95.26%	4.13%
		E2		23		
DB3	S1–S11	E1		17	90.81%	22.25%
DB6	S1–S10	D1-T1		7	96.72%	2.68%

**Table 4:** Comparison of the recognition effects with those of other methods.

Authors	No. of Subjects	Subject Type	Data	Class	Acc
Sun, Song [25]	40	Healthy	DB2	34	89.84%
Murugiah, Jino [27]	40	Healthy	DB2	50	90.08%
Pancholi, Jain [28]	40	Healthy	DB2	49	88.88%
	5	Amputee	DB3	49	81.67%
de Souza, Bloedow [29]	10	Amputee	DB3	50	56.08%
Khorram, Lin [30]	10	Healthy	DB6	7	95.56%
This work	20	Healthy	DB2	40	95.26%
	11	Amputee	DB3	17	90.81%
	10	Healthy	DB6	7	96.72%

**Table 5:** The gesture recognition accuracy for 5 experimenters.

	Gender	Age	Number of Gestures	Accuracy
S1	Male	25	6	97.67%
S2	Male	25		92.33%
S3	Female	26		98%
S4	Male	24		98%
S5	Male	25		99%

## Data Availability

The electromyographic signal processing method and the trained model proposed in this paper are available at the following URL: https://github.com/an0724/MM-DNN, accessed on 12 January 2026.

## Appendix A

The magnitude of the sEMG signal data was very small. Without considering the effect of extreme values, we set the sEMG signal data as z. The maximum value is denoted by $z_{\max}$, and the minimum value is denoted by $z_{\min}$. The ratio of the maximum and minimum values, j, is computed as $j=\frac{z_{\max}}{z_{\min}}$. After the sliding average power is applied to the sEMG signals, the square will change the magnitude difference. The maximum value of the sEMG signal data is $z_{\max}^{2}$ and the minimum value is $z_{\min}^{2}$. The ratio of the maximum and minimum values, $j_{s}$, is calculated as $j_{s}=\frac{z_{\max}^{2}}{z_{\min}^{2}}=j^{2}$. Then, the lg operation is performed; the maximum value of the data is $\mathrm{lg}z_{\max}^{2}$ and the minimum value is $\mathrm{lg}z_{\min}^{2}$. The difference J between the maximum and minimum values is calculated as in Equation (A1):

(A1) $$J=\mathrm{lg}z_{\max}^{2}-\mathrm{lg}z_{\min}^{2}=\mathrm{lg}\left(\frac{z_{\max}^{2}}{z_{\min}^{2}}\right)=2\mathrm{lg}j$$

From Equation (A1), the theoretical span value of $x_{i}^{L}$ is J. Because the sigmoid function is symmetric about the origin, its middle segment changes relatively rapidly, so the original sEMG signals are deflated to an interval symmetric about the origin $\left[-J/2,J/2\right]$.
